# Destructive and Non-Destructive Methods for aDNA Isolation from Teeth and Their Analysis: A Comparison

**DOI:** 10.3390/genes16091059

**Published:** 2025-09-09

**Authors:** Agnieszka Dobosz, Anna Jonkisz, Arleta Lebioda, Jerzy Kawecki, Tadeusz Dobosz

**Affiliations:** 1 Division of Basic Medical Sciences, Department of Basic Medical Sciences and Immunology, Wroclaw Medical University, Borowska 211, 50-556 Wrocław, Poland; 2 Molecular Technique Unit, Department of Forensic Medicine, Wroclaw Medical University, Marii Curie-Skłodowskiej 52, 50-367 Wroclaw, Poland; 3 Forensic Medicine Institute, Department of Forensic Medicine, Wroclaw Medical University, Mikulicza-Radeckiego 4, 50-368 Wroclaw, Poland

**Keywords:** ancient DNA, non-destructive, root-canal, tooth, silica purification, forensic dentistry

## Abstract

Background/Objectives: DNA analysis can be used to expand our understanding of extinct populations and the history of the world and humankind. Dental cavities often contain uncontaminated remains of ancient DNA (aDNA). Archaeological excavations are a convenient source for various samples; however, in almost all extraction methods, a piece of bone or tooth is powdered before extraction, thereby causing damage to archaeological samples that are often irreplaceable and unique. This study aimed to develop a method that enables the collection of DNA from teeth without causing significant damage. Methods: This study presents two methods of DNA extraction from teeth: destructive and non-destructive. Both contemporary and archaeological teeth were examined using both destructive and non-destructive approaches to compare their efficiency. To verify the results, methods such as quantitative RT-PCR, STR analysis, and Y-SNP analysis were employed. Results: Extraction efficiency plays a critical role in this field of research. The main steps of the DNA extraction method were compared and optimized based on purification and using quantitative PCR. Conclusions: The results demonstrate that a non-destructive method of DNA isolation from human teeth can be used successfully, especially when teeth are unique and cannot be destroyed during the examination process. This method yields an appropriate amount of DNA for sequencing.

## 1. Introduction

Several pioneering papers have presented methods for non-destructive DNA extraction. The first mention of such a method appeared in a patent No. WO2007093448A1 [1], which described the method of DNA extraction from plant root border cells. The investigations of Thomsen et al. in 2009 [2] are also important. The authors extracted DNA from museum insect specimens. The authors flushed insects in an aggressive solution to obtain DNA from them. After that, the insects were empty inside. In 2015, Guzman-Larralde et al. compared six methods of non-destructive DNA preparation from insects [3].

In 2003, Wasko et al. investigated fish, obtaining DNA from fins and scales in a non-destructive manner [4]. In 2017, Shepherd [5] wrote about a non-destructive DNA sampling technique for herbarium specimens. Li et al. [6] developed a micro needle-based non-destructive method for testing individual seeds. In 2012 and 2022, two teams, Bolnick et al. [7] and Rayo et al. [8], published papers on the non-destructive sampling of human skeletal remains and the non-destructive extraction of DNA from preserved tissues in medical collections, respectively.

Bones and teeth are the longest-lasting physical evidence of human or animal presence at an archaeological site. They are also the most widely used sources of samples for ancient DNA (aDNA) studies. In skeletons, the most extended survival of material DNA after death is associated with retarded rates of decomposition, resulting from the adsorption of DNA to hydroxyapatite [9,10], low water content, ”mummification” of individual cells, and physical exclusion of microbes and external contaminants [9]. Studies by Pruvost et al. [11] reveal that DNA preservation is better in freshly excavated untreated bones, and that post-excavation treatments and/or storage conditions negatively influence DNA yields and quality.

An awareness of sample handling as a source of contamination has led researchers to investigate teeth as a source of ancient DNA. One hypothetical benefit is protection conferred by the enamel [9,12]. Several studies have reported better aDNA yields in teeth than in bone [9,12], allowing these parameters to be correlated with modern DNA contamination in both bones and teeth. It is widely acknowledged that human bones differ in their suitability for research purposes. Our experience shows that the petrous bone yields the maximal amount of DNA. However, compared to teeth, it has a bigger proportion of bacterial and fungal DNA (Pinhasi et al. [13] set out that only 1% of the obtained aDNA is human). On the contrary, Drancour et al. showed that DNA obtained from teeth is relatively lightly contaminated [14].

The history of non-destructive DNA isolation from teeth is short. The first approach was carried out by Cobb in 2002 [15]. In this paper, the author obtained DNA using powder from drilled nerve canals. The next approach was carried out by Rohland et al. [16]. The authors immersed teeth in an aggressive solution from which DNA was obtained. In 2021, their method was improved and developed by Harney et al. [17]. Rehman et al. (2025) [18] emphasized the importance of non-destructive DNA isolation from teeth.

The next approach was undertaken by Hervella et al. [19], who drilled into the tooth chamber using a slightly damaged area of the tooth. All the mentioned methods have disadvantages. The first method destroyed the surface of the tooth, the second destroyed nerve canals, and the third led to the appearance of changes resembling carious lesions. In 2023, Wei et al. isolated DNA from tooth pulp and hard tissue, obtaining similar results [20].

Analysis by Malaver and Yunis [21] reveals that in cases of advanced decomposition of corpses, it is possible to attempt to extract DNA from cement and dentin. The results of amplifying the HV2 region of the mitochondrial DNA showed that dentin and cement acted as protective factors for the cells, allowing the conservation of the DNA.

In this study, the authors present an innovative, non-invasive method for tooth sampling. Also, the influence of various chemicals added to the extraction buffer and the factors such as extraction temperature and extraction duration on DNA extraction was evaluated. This paper aimed to compare two methods of DNA extraction to determine which one is more effective and to verify whether the non-destructive method can provide sufficient DNA for all standard molecular biology techniques.

## 2. Materials and Methods

### 2.1. Samples

Thirty-six teeth were obtained from eight unidentified bodies during the autopsy of NN (lat. nomen nescio) persons, after obtaining permission from the authority office, in the Department of Forensic Medicine, Wroclaw Medical University, in 1992–1993. The estimated age at death of these corpses ranged between 25 and 60 years (median, 42.5 years). The teeth were stored at room or −20 °C temperatures and studied after 0.5 to 20 years from autopsy. Additionally, when the method seemed ready to use, another 70 museum, archaeological, and post-exhumation teeth were used; the oldest was approximately 50,000 to 100,000 years old. The list of samples and their origins is shown below:

No. 1. Molar teeth of an NN male aged about 50, obtained on autopsy of a severely putrefied cadaver at the Forensic Medicine Department in Wrocław (*N* = 6).
No. 2. Molar teeth of an NN female aged about 25, obtained on autopsy of a severely putrefied cadaver at the Forensic Medicine Department in Wrocław (*N* = 6).
No. 3. Molar teeth of an NN male aged about 30, obtained on autopsy of a severely putrefied cadaver at the Forensic Medicine Department in Wrocław (*N* = 6).
No. 4. Molar teeth of an NN male aged about 45, obtained on autopsy of a severely putrefied cadaver at the Forensic Medicine Department in Wrocław (*N* = 6).
No. 5. Teeth from the Museum of Forensic Medicine, stored at RT (room temperature) for about 60 years (*N* = 4).
No. 6. Molar teeth of an NN male aged about 60, obtained on autopsy at the Forensic Medicine Department in Wrocław (*N* = 4).
No. 7. Skull after preparation with partly preserved dentition. Stored at room temperature (*N* = 2).
No. 8. Molar teeth of an NN male (accurate evidence material) (*N* = 2).
No. 9. Molar teeth of an NN male aged about 50, obtained on autopsy of a severely putrefied cadaver at the Forensic Medicine Department in Wrocław, 20 years stored at RT (*N* = 6).
No. 10. Molar teeth of an NN female aged about 25, obtained on autopsy of a severely putrefied mummified cadaver at the Forensic Medicine Department in Wrocław, 20 years stored at RT (*N* = 6).
No. 11. Molar teeth of an NN male aged about 30, found in fresh water, obtained on autopsy of a severely putrefied cadaver at the Forensic Medicine Department in Wrocław, 20 years stored at RT (*N* = 6).
No. 12. Molar teeth of an NN male aged about 45, obtained on autopsy of a severely putrefied cadaver at the Forensic Medicine Department in Wrocław, 20 years stored at RT (*N* = 6).
No. 13. Molar teeth of an NN male aged about 30, obtained on autopsy of a completely skeletonized cadaver at the Forensic Medicine Department in Wrocław, 20 years stored at RT (*N* = 4).
No. 14. Molar teeth of an NN male aged about 60, obtained on autopsy at the Forensic Medicine Department in Wrocław, 20 years stored at RT (*N* = 4).
No. 15. Molar tooth from the Museum of Forensic Medicine, 55 years old, stored at RT (*N* = 1).
No. 16. Museum skull after chemical whitening and preparation, about 60 years old, male, with partly preserved dentition. Stored at RT (*N* = 2).
No. 17. Teeth from exhumation of the victim of domestic war, soil grave, 60 years old (Opole, Poland), stored at RT (N = 2).
No. 18. A partially burned tooth from World War II, approximately 65 years old, stored at RT (*N* = 1).
No. 19. Teeth of an aristocratic Lithuanian family, XVII Century (1562–1620), buried in a common grave in a secret place, after the robbery and devastation of the Grave Chapel by soldiers about 30 years before the last burial (Dubingiai, Lithuania) (*N* = 13).
No. 20. Molar tooth from a soil grave, XV Century (Wroclaw, Poland), stored at RT (*N* = 1).
No. 21. Tooth from the Catholic Church Relic, 13th Century (Kraków, Poland), stored at RT (*N* = 1).
No. 22. Teeth from archeological excavations at the site of Milicz churchyard (XI ng/μL Century). Since the 60s, the material has been stored at room temperature at the Bone Collection of the Museum of Human History, Anthropology Institute, Polish Academy of Sciences in Wrocław (*N* = 5).
No. 23. Molar teeth, X Century (Ryczyn, Poland), stored at RT (*N* = 4).
No. 24. Molar tooth of the migration period, 5th century (Kroczycka Cave, Poland), stored at RT (*N* = 1).
No. 25. Neolithic, molar teeth 4000 BC; Jagodno, Wroclaw, Poland [22] (*N* = 2).
No. 26. Neanderthal molar tooth, dated approximately 100,000 years (Stajnia Cave, Poland), obtained DNA was repaired and cloned before testing; however, only male AMEL was detected [23] (*N* = 1).

### 2.2. Destructive Approach

Thirty-six human teeth from autopsies of NN persons with unknown personality were tested. Each tooth was carefully washed for approximately 2 min in cold tap water, then gently mechanically cleaned using a small piece of glass paper (100 to 200 grit grade) and flushed for 1 h in deionized water. If many persons had touched the tooth, the next step was to UV-irradiate both sides of the tooth for 1 min, using radiation energy of 3 J/cm^2^, in a VILBER-LOURMAT Biolink^TM^ BLX UV Crosslinker (Fisher Scientific, Schwerte, Germany). Next, the teeth were crushed in liquid nitrogen, using the SPEX SamplePrep 6870 Freezer Mill (SPEX SamplePrep LLC, Metuchen, NJ, USA). Powder was solubilized using 0.75 to 1.25 mL (depending on tooth dimensions) of the extraction buffer containing 0.5 M EDTA pH 8.0, 50 mM DTT, 0.5% Tween^®^20, and 20 μL proteinase K (0.25 mg/mL) was added and incubated at 56 °C for 24 to 48 h to complete solubilization of the powder. The crude fluid containing DNA was concentrated and purified on a silica bed, according to Rohland and Hofreiter [24], using a commercial silica kit QIAamp DNA Micro Kit (Qiagen Wrocław, Wrocław, Poland) or (only after extraction from molar tooth) a phenol–chloroform protocol, according to Chomczynski and Sacchi [25] with modifications, followed by QIAquick PCR Purification Kit (Qiagen Wrocław, Wrocław, Poland).

### 2.3. DNA Extraction Methods

In both DNA extraction methods (non-destructive and destructive), strict contamination precautions were taken as prescribed for aDNA research according to Cooper and Poinar [26], Hebsgaard et al. [27], Stoneking [28], and Willerslev and Cooper [29]. Protective clothing was worn, consisting of latex gloves, a hairnet, a face mask, scrubs, and booties. Before wearing, all clothing was autoclaved. Tooth sampling was performed in an area of the laboratory dedicated to aDNA research, under a sterilized laminar flow hood to protect the working surface. The sampling area was sterilized with 0.6% sodium hypochlorite, followed by 70% ethanol (according to Gibbon et al. [30] and Kolman and Tuross [31]). In the destructive method, only intact (i.e., without caries and requiring no stomatological intervention) human teeth were selected for each subject.

Contaminating modern DNA was removed from the surface of the teeth within 1 h of sanding, using laboratory-grade quartz or broken glass sand (from various supplies), and each lot was checked for human DNA content. Selected teeth (often handled without gloves) were briefly UV-irradiated on both sides for 1 min, using a radiation energy of 3 J/cm^2^ (Kalmár et al. [32], with modifications).

In the destructive method, the tooth was placed into liquid nitrogen for 5 min, and then crushed in a Spex. The obtained powder was flooded with extraction buffer (0.5 M EDTA (pH 8.0), 50 mM DTT, 0.5% Tween^®^20, and 20 μL of proteinase K (0.25 mg/mL) for 12 h.

**Figure 1:**
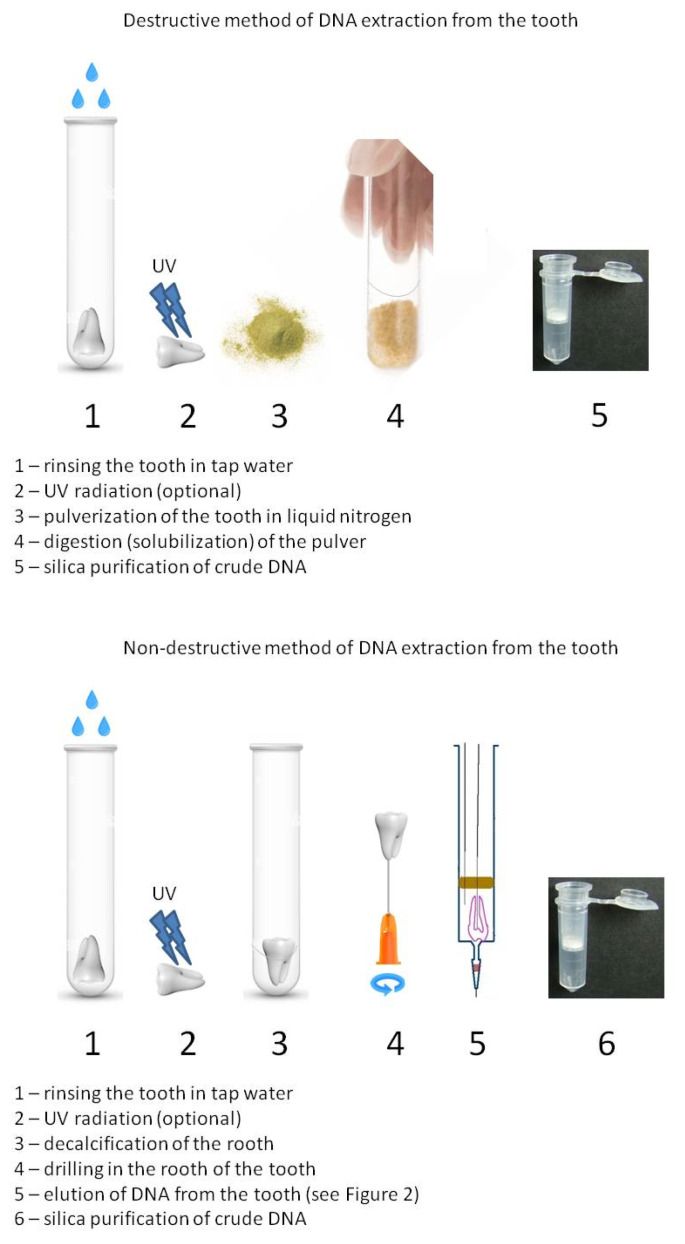
Comparison between destructive and non-destructive methods of DNA extraction from the tooth. The complete system for non-destructive DNA elution from human tooth is shown in Figure 2.

The comparison of destructive and non-destructive DNA extraction methods is presented in Figure 1.

**Figure 2:**
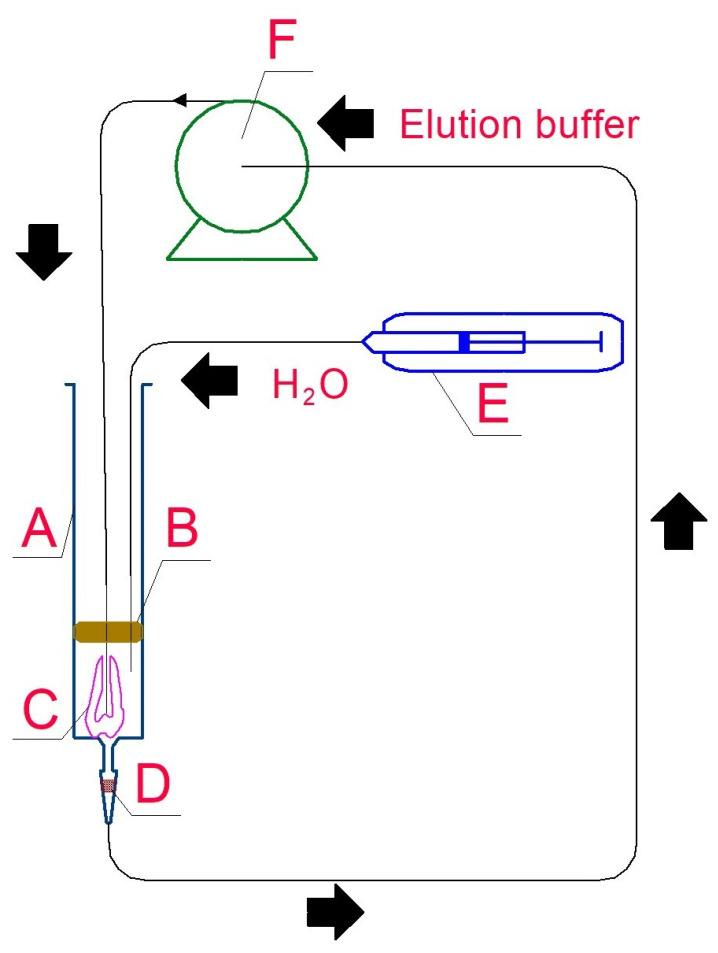
Complete system for non-destructive DNA elution from human tooth. A—three-part syringe 2, 5, 10 or 20 mL (according to tooth diameter), B—rubber piston with removed plastic part, perforated by two needles, 0.4 and 0.8 mm, attached to silicone or polyethylene tubings, C—tooth with an open nerve canal and an introduced 0.4 mm needle, D—filter tip (100 μL) with an anticontamination filter; check if the lot does not bind DNA, E—linear syringe pump with a 1 mL syringe filled with 18.6 MΩ water with the addition of two portions of proteinase K, F—peristaltic pump adjusted to flow 2 mL/h.

In the non-destructive method, the first step of DNA extraction was gentle, short final decontamination by final flushing of the tooth surface with 5 mL of 0.5 M EDTANa_2_ buffer, pH 8.0, and 0.5% Tween^®^20 [33] followed by incubation of the tooth root in saturated EDTANa_2_ adjusted to pH 8.0 by EDTANa_4_, for two to twelve hours, at 37 °C, mixing sporadically. Next, access to the dental cavity was opened over the nerve canal by hand, using gentle boring with a thin 0.5 × 25 mm needle. Next, a 0.4 × 35 (or longer) needle with a blunt end was introduced into silicone tubing, which was perforated through the rubber syringe piston and introduced into the dental cavity. The complete elution system is presented in Figure 2.

The syringe with filter was placed in the thermostat for incubation at 37 °C or 56 °C for 12 to 24 h. Higher temperatures seemed beneficial because the DNA yield was consistently higher, and these temperatures also prevent bacterial growth. However, on the other hand, proteinase K is thermosensitive. Using syringe ”E”, molecular biology-grade water was introduced into the system to observe the first sign of fluid circulation. Next, 250 to 500 μL (depending on tooth dimensions) of the extraction buffer, containing 0.5 M EDTA (pH 8.0), 50 mM DTT, 0.5% Tween^®^20, and 20 μL of proteinase K (0.25 mg/mL), was introduced into the system using the same syringe. Finally, these syringes (filled with water twice the volume of the extracting buffer) were placed into a syringe pump, programmed to empty the syringes in 12 h of work. The circulating buffer rinsed the dental root canal and dental chamber, but a supply of additional water was necessary, resulting in water evaporation, despite a theoretically hermetic circulation system.

In both extraction methods, after elution, DNA was concentrated and purified on a silica bed, according to Rohland and Hofreiter [24], using a commercial silica kit QIAamp DNA Micro Kit (Qiagen) or (after extraction from relatively fresh molar) twice, by phenol–chloroform protocol according to Chomczynski and Sacchi [25] with minor modifications, followed by QIAquick PCR Purification Kit (Qiagen). The average final silica bed elution volume was 50 microliters; however, the typical yield and concentration of DNA varied (see Section 3). In the non-destructive method, different combinations of incubation temperatures during digestion, components of the extraction buffer, binding times, and concentrations of K proteinase were compared.

### 2.4. Purification of Crude DNA

Three methods of DNA purification were used. First, a contact was made with the crude lysate usng chloroform and isoamyl alcohol. Then, two methods of purification of crude DNA were used: the DNA Extraction Kit FERMENTAS and the QIAmp DNA Micro Kit (Qiagen), according to the protocols provided by the manufacurers.

### 2.5. Quantitative RT PCR

To assess the human DNA yields present in a sample, a probe-based quantitative PCR system, Quantifiler^®^ Human DNA Real-Time Quantification Kit (ABI Prism^®^7700; Applied Biosystems, Foster City, CA, USA), was used. A dilution series of a PCR product obtained previously as a standard, according to the protocol, was used, along with a commercial standard and a genomic standard prepared and validated in our laboratory.

### 2.6. STR Analysis

To detect possible contamination by exogenous modern DNA and verify the reproducibility of the results, STR and Y-SNP analyses were employed. Amplifications were performed with the tests based on SGM Plus (for the destructive method) and NGM Select (for the non-destructive method), which amplify simultaneously ten STRs: D3S1358, vWA, FGA, D8S1179, D21S11, D18S51, D5S818, TH01, D16S539, D2S1338, D19S433) and the amelogenin locus (determining the individual’s sex) and 16 loci D10S1248, D3S1358, vWA, D16S539, D2S1338, amelogenin, D8S1179, D21S11, D18S51, D19S433, TH01, FGA, D22S1045, D2S441, D1S1656, and D12S391), with additional primers for the SE33 locus. Each amplification was carried out with a 50 μL reaction mixture containing 25 μL Multiplex PCR Reaction Mix (Qiagen), 5 μL primer mix, H_2_O, and 4 μL of ancient DNA sample. Cycling parameters were 95 °C for 15 min, followed by 35 to 50 cycles with the following temperatures and times: 94 °C, 30 s; 57 °C, 90 s; 72 °C, 1 min and a final delay of 45 min at 60 °C. Standard kits, not miniSTR kits, were used to unify the methods used in our laboratory. The PCR products were analyzed by capillary electrophoresis using an automated sequencer, the ABI Prism 310 or 3130, with the internal size standard GeneScanTM 500 LIZ^®^ Size Standard (Applied Biosystems).

### 2.7. Y-SNP Analysis

Y-chromosome genotyping was performed using primers [34] by multiplex minisequencing with the ABI Prism^®^ SNaPshot Multiplex Kit (Applied Biosystems), preceded by multiplex PCR using the Qiagen^®^ Multiplex PCR Kit (Qiagen), according to the manufacturer’s protocol. The products of minisequencing were separated by capillary electrophoresis, along with GeneScan™-120LIZ^®^ Size Standard (Applied Biosystems), on an ABI Prism 310 or 3130 Genetic Analyzer (Applied Biosystems), for genetic analysis as well as GeneMapper ID v3.2 (Applied Biosystems). The genotypes of all individuals involved in collecting and researching the samples were determined and compared to the results obtained from the ancient bone samples. Moreover, DNA was extracted from each sample at least twice, and from each extract, at least three PCR amplifications were made to assess the results.

The SNPs from the Y chromosome were the shortest amplicons, and the rate of success was higher than that of STR.

## 3. Results

**Figure 3:**
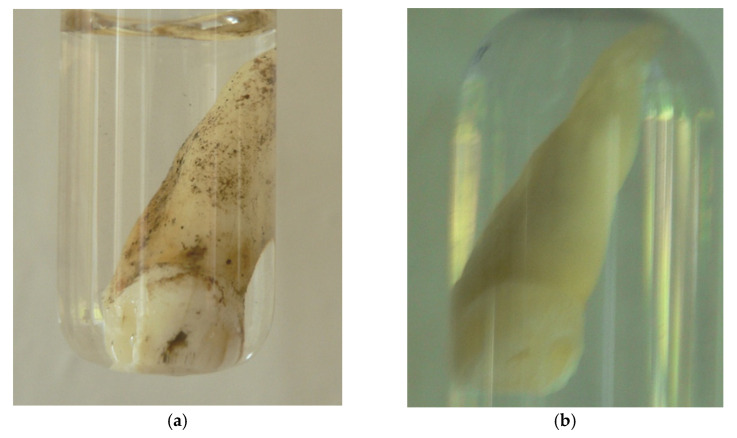
Human tooth before (**a**) and after (**b**) non-destructive DNA isolation.

The presented method of DNA extraction from teeth enabled preservation of the material in a practically unchanged condition. After the DNA isolation process, the teeth were protected from any contamination from the background. In the case of contemporary teeth, bleaching of the enamel was observed. An example of the tooth before and after nondestructive DNA isolation is presented in Figure 3. For better durability, the tooth was placed in a water bath three times (each for 2 h at room temperature) after drying. It was then gently greased with paraffin oil, vaseline, or lanolin (after room temperature drying) to prevent cracking of the roots. Because the dental plaque was removed during aDNA preparation, it was scraping it before starting was recommended, especially in the cases when calculus plate was tested.

**Figure 4:**
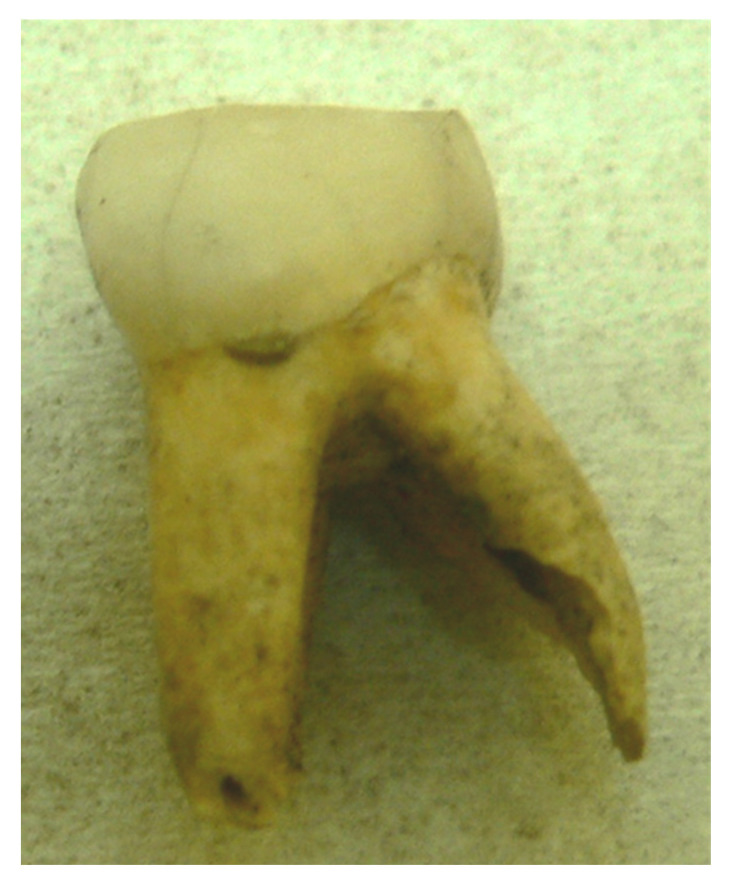
A 900-year-old tooth with visible minor damage to the root apex.

The only damage to the investigated teeth concerned the root at the entrance of the dental canal and was associated with the insertion of the needle, as pictured in Figure 4. The visible root damage was made during the attempt to drill in the nerve channel without decalcification.

The first step of the investigation was DNA quantification using a destructive method of DNA extraction. Thirty-six fresh autopsy teeth were examined, and the results are presented in Table 1. Table 1 contains the source and age of the teeth, temperature of elution, method of purification, total yield of DNA, and average DNA concentration with SD (quantification).

The results presented in Table 1 show that the number of useful amplicons regularly decreased with increasing time between death and DNA isolation, with rare exceptions. From Table 1, it is also clear that the results of DNA profiling are unpredictable, and the remaining DNA concentration should be used only as a guide for determining the amount of DNA to be used for the PCR test. In two cases (3.6%), no DNA profiles were obtained. In 10 teeth (17.9%), partial profiles (without some of the largest amplicons, approximately 100 to 300 bp) were obtained. In 44 cases (78.5%), complete, clear DNA profiles were obtained. Using three very fresh human wisdom teeth extracted for stomatological reasons, about two micrograms of DNA was received, which seems to be the maximum possible yield from complete human tooth DNA (from pooled dentine, dental pulp, and nerve). Due to the significant fragmentation of the DNA, the PCR and SNaPshot primers were designed to enable the maximal shortening of the analyzed amplicons. Three ranges of amplicons, 100 bp, 200 bp, and 300 bp, were identified.

The average efficacy of destructive DNA tooth isolation reached approximately 95% with amplicons of 50 bp, 82% with amplicons of 100 bp, and 79% with amplicons of 300 bp.

The next step in the investigations was carried out with a non-destructive approach to DNA isolation. To evaluate the efficiency of non-destructive DNA extraction methods, the Real-Time PCR reaction was performed using the Quantifiler^®^ Human DNA Quantification kit (Applied Biosystems), and the average DNA concentrations are presented in Table 2. Because the AMEL and SNP loci had the smallest amplicons, and the efficacy of determining these loci was the highest. The performance of PCR amplification, both with and without bovine serum albumin (BSA), was assessed, yielding the same results. The average DNA yield ranged from 0.01 to 0.4 μg, with a typical concentration of approximately 0.1 to 0.4 ng/μL.

**Figure 5:**
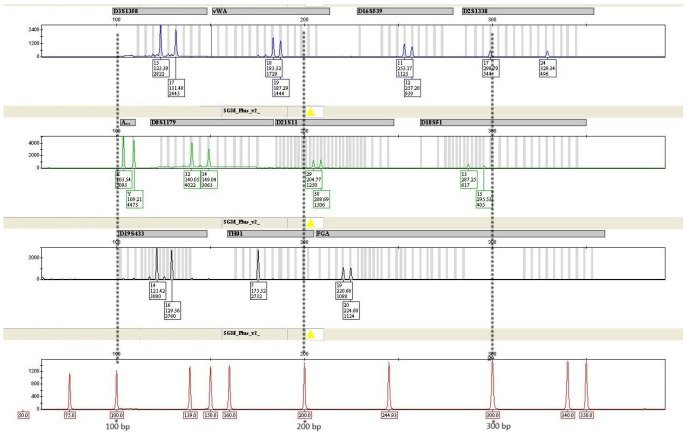
The results of STR typing of DNA from a human tooth. Complete profile of sample No. 15. The intervals 100, 200, and 300 bp have been marked with asterisks.

**Figure 6:**
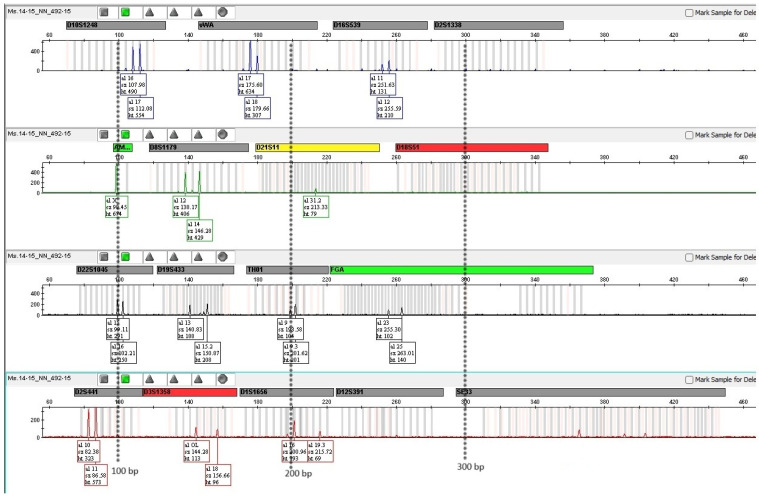
The results of STR typing of aDNA from a human tooth. Incomplete profile of sample No. 20 (15th-century). The intervals 100, 200, and 300 bp have been marked with asterisks.

Table 2 presents the temperature of the elution process, the average amount of obtained aDNA, detailed data on the aDNA yield, and the results of DNA profiling. The obtained human aDNA concentration varied significantly, depending on environmental conditions, age, tooth type, and method of crude lysate purification, and ranged from 0.02 ng/μL to 9.37 ng/μL. Sample No. 8 was the control (fresh autopsy teeth), prepared using a non-destructive approach, and the control DNA concentration was 14.92 ng/μL. A complete DNA profile was obtained in 26% of the DNA tested, and 74% showed damage to longer amplicons. Figure 5 shows the results of DNA profiling in the case of a 55-year-old tooth from a museum collection. It illustrates the situation where a complete DNA profile can be read with success. Figure 6 shows incomplete results obtained from a 15th-century tooth from an archaeological soil grave. Only amplicons of 200 bp or less are visible.

Examining the data presented in Table 1 and Table 2 reveals the same trends in the method of DNA extraction from teeth. First, the influence of extraction temperature (37 °C vs. 56 °C) on the effectiveness of DNA extraction can be analyzed. According to the data, it was calculated that at both temperatures, the average rate of success was about 83%; however, their efficiencies varied greatly (0.11 ng/μL at 37 °C vs. 1.44 ng/μL at 56 °C). The influence of the purification method on DNA extraction was also analyzed. The classic phenol–chloroform purification method yielded 75% efficiency compared to 87% when using silica kits for purification.

The third parameter considered was the age of the sample. All samples were categorized by age, as shown in Table 3.

Table 3 shows that the efficiency of DNA testing depends primarily on the storage conditions, rather than the age of the teeth. The rate of success was calculated based on autosomal STR typing. Three thresholds were assigned: 100 bp, 200 bp and 300 bp. Incomplete results (IR) (only 200 bp and 100 bp) were also treated as “success”, because some results were obtained. The average success rate in extracting DNA from fresh teeth using a destructive method was 87%; when using a non-destructive method, the average success rate decreased to approximately 85.5%. The average success rate between the destructive and non-destructive approaches is small, at approximately 1.5%.

## 4. Discussion

Gomes et al. [35] compared the destructive and non-destructive approaches to DNA isolation from human teeth, similar to the ones presented in this paper. Contrary to the results presented here, they found that the non-destructive approach yielded lower DNA quantity.

In the studies presented in this paper, the average efficacy of non-destructive DNA tooth isolation was decreased compared to destructive methods, reaching approximately 80% with amplicons of 100 bp, 61% with amplicons of 200 bp, and 31% with amplicons exceeding 300 bp. As can be seen from the results, the correlation of the DNA yield obtained from the teeth of the same person, using the same method of isolation, showed differences. The reason could be the natural diversity, decay stage, or differences in the mass of the dental pulp between the investigated teeth.

The aim of this work was also to show the influence of different physical parameters on the productivity of DNA extraction. The time between death and DNA isolation is not as crucial as environmental storage conditions, but it may provide preliminary information about the likelihood of success. Suppose Allentoft et al. [36] are correct, and the half-life of aDNA is approximately 500 years. In that case, the theoretical time limit for successful PCR of amplicons about 300 bp in length from a single human tooth is commonly no longer than 5000 years. On the other hand, shorter amplicons (for example, Y-SNP with amplicons about 50 bp) may be successfully determined in older material. During investigations of aDNA, two questions should be considered: the first is connected with authentication, and the second with DNA damage patterns. For this reason, the DNA of the Neanderthal had to be repaired before examination, which was necessary for determining the sex of the sample [23].

Considering the environmental influences on DNA extraction, an acidic pH appears to be the most significant. Bacteria and fungi influence another problem. The teeth collected for examination are usually not sterile. Microbes may survive after irrigation, so lysis at higher temperatures is recommended. However, the use of higher temperatures to inhibit the development of bacterial flora is limited by the sensitivity of damaged DNA to temperature. Additionally, due to the low thermostability of proteinase K above 50 °C, it is recommended to add the next portion of proteinase K after each 12 h lysis. Microbiological contaminations often cause difficulties in PCR [37,38], especially when testing DNA that is strongly degraded [26,39]. UV sterilization should not be used, as it typically disrupts DNA strands by breaking the covalent bonds between bases, thereby impairing PCR [35]. The presented isolation method enables the extraction of fossil DNA from the pulp chamber without the need for preliminary aggressive sterilization.

Decalcification of teeth is not essential to obtain DNA. It also prolongs the process and increases its costs. However, the majority of authors claim that the initial decalcification significantly affects DNA purity, with minimal loss in its amount [40,41]. This problem was resolved by Essel et al., who trapped DNA, resulting in a 30-fold increase in the amount of DNA obtained [42].

Reducing agents (e.g., 50 mM dithiothreitol) and detergents (e.g., Tween^®^20) should be added to the extraction solution to denature proteins and disrupt intact cell membranes when extracting modern or historical bones or teeth. N-phenacylthiazolium bromide (PTB) can be added to cleave Maillard products (sugar-derived protein crosslinks) [24]. For samples that do not contain PCR inhibitors, salts other than guanidinium thiocyanate (GuSCN) may be used, for example, NaCl [43]. These non-chaotropic salts are significantly cheaper and perform equally well, if not better, in terms of DNA recovery. To increase the concentration of the obtained DNA, an additional portion of proteinase K should be added after 12 h of lysis at 56 °C.

Results of quantitative PCR show that the extraction buffer, consisting of 0.5 M EDTA-Na_2_, Tween^®^20, DTT, and proteinase K, is the most effective technique for digesting teeth and binding DNA to silica (either laboratory-made or commercial kit) for isolating ancient DNA. A laboratory-made silica suspension is the most cost-effective and efficient method for DNA recovery. However, the stages of its preparation are burdened with additional risk of contamination, which is absent in the case of the commercial kits QIAamp DNA Micro Kit (Qiagen) or Fermentas. Also, it was observed that a two-fold prolongation of DNA binding time to silica resulted in an increased efficacy of the extraction process.

The use of phenol–chloroform extraction can sometimes lead to losses in DNA recovery and result in less efficient purification, making it a consideration to avoid in studies involving fossil material.

The effect of temperature on the amount of DNA obtained was also analyzed. Contrary to earlier results of Rohland and Hofreiter [24], the investigations presented in this paper demonstrated a weak correlation between increased extraction temperature (up to 56 °C) and increased DNA concentration. Moreover, increased extraction temperature inhibits the development of the majority of microorganisms and decreases the amount of PCR inhibitors. The influence of temperature on DNA extraction was also examined by Essel et al. [44], who highlighted the critical role of temperature in the isolation process.

The genetic analysis of nuclear DNA was based on STR and SNP loci. Due to the significant fragmentation of the fossil DNA, the PCR and SNaPshot primers were designed to enable the maximal shortening of the analyzed amplicons. The majority of the SNaPshot reaction products do not exceed 100 bp, whereas the PCR product is 250 bp. This is a common occurrence in degraded DNA; however only confirmed results are accepted in our laboratory.

The obtained results of DNA loci STR and SNP amplification confirm the usefulness of this analysis in evaluating fossil DNA, as previously observed by Zierdt et al. [45], Burger et al. [46], Ricaut et al. [47], and Dixon et al. [48]. It allows for confirmation on a molecular basis of the gender that had been determined based on morphological traits.

Correlation of STR and Y-SNP locus amplification from six independent DNA isolations enables verification of the analysis and identification of possible contaminations. Due to significant DNA degradation, some of the STR loci were shortened, producing more than two peaks in the electrophoretic image.

The presented extraction method was successfully used in the examination of authentic fossil material [23,49]. The investigation conducted in our laboratory showed that in the analysis of fossil DNA from significantly degraded material, teeth are a preferred tissue choice. If Next Generation Sequencing (NGS) is planned, the type of sequencer should be checked to determine whether short amplicons are suitable for it. Our laboratory has no experience with NGS, but it is a powerful and promising tool in molecular biology. For example, in paper of Tin et al. [50], whole mtDNA was obtained using NGS, while Juras et al. used NGS to confirm the method of direct maternal relationship [51].

The non-destructive method of DNA isolation from human teeth is technically mature, suitable for museum collections, and accepted by various religions, making it suitable for widespread use in criminalistics, anthropology, evolutionary studies, relic research, and archaeology. The non-destructive approach for aDNA isolation from teeth has two disadvantages and one significant advantage. Firstly, the method is more laborious and costly, and the success rate is about 1.5% (compared to the destructive method). Still, in some cases, this is the only possible method for processing the very valuable teeth.

## Figures and Tables

**Table 1:** Description of autopsy material used in the studies and the obtained findings (destructive method).

No. of Sample (See List in Paragraph Section 2.1.)	Temp. of Elution	Method of DNA Purification from Crude Extract	DNA Yield (μg)	Concentration of Prepared DNA (ng/μL)	DNA Average Concentration, (ng/μL) ± SD
No. 1	37 °C	silica suspension	0.0015	0.03	0.04 ± 0.02
	56 °C	silica suspension	0.004	0.08	
	37 °C	QIAamp DNA Micro Kit (Qiagen)	0.002	0.04	
	56 °C	QIAamp DNA Micro Kit (Qiagen)	0.0025	0.05	
	37 °C	phenol–chloroform	0.001	0.02	
	56 °C	phenol–chloroform	0.001	0.02	
No. 2	37 °C	silica suspension	0.01	0.20	0.36 ± 0.12
	56 °C	silica suspension	0.027	0.53	
	37 °C	QIAamp DNA Micro Kit (Qiagen)	0.02	0.40	
	56 °C	QIAamp DNA Micro Kit (Qiagen)	0.02	0.40	
	37 °C	phenol–chloroform	0.013	0.26	
	56 °C	phenol–chloroform	0.018	0.35	
No. 3	37 °C	silica suspension	0.0025	0.05	0.03 ± 0.03
	56 °C	silica suspension	0.003	0.06	
	37 °C	QIAamp DNA Micro Kit (Qiagen)	0.001	0.02	
	56 °C	QIAamp DNA Micro Kit (Qiagen)	0.0015	0.03	
	37 °C	phenol–chloroform	0	0.0	
	56 °C	phenol–chloroform	0	0.0	
No. 4	37 °C	silica suspension	0.005	0.10	0.09 ± 0.02
	56 °C	silica suspension	0.006	0.12	
	37 °C	QIAamp DNA Micro Kit (Qiagen)	0.0045	0.09	
	56 °C	QIAamp DNA Micro Kit (Qiagen)	0.005	0.10	
	37 °C	phenol–chloroform	0.003	0.06	
	56 °C	phenol–chloroform	0.003	0.06	
No. 5	56 °C	0.5 M EDTANa_2_ pH 8.0 + 0.5% Tween^®^20 + 50 mM DTT + proteinase K	0.076	1.52	1.11 ± 0.29
		0.5 M EDTANa_2_ pH 8.0 + 0.5% Tween^®^20 + proteinase K	0.052	1.03	
		0.5 M EDTANa_2_ pH 8.0 + proteinase K	0.044	0.87	
		0.5 M EDTANa_2_ pH 8.0 + 20% SDS + 1 M Tris-HCl. 5 M NaCl + proteinase K	0.05	1.0	
No. 6	56 °C	0.5 M EDTANa_2_ pH 8.0 + 0.5% Tween^®^20 + 50 mM DTT + proteinase K	0.017	0.33	0.21 ± 0.09
		0.5 M EDTANa_2_ pH 8.0 + 0.5% Tween^®^20 + proteinase K	0.011	0.22	
		0.5 M EDTANa_2_ pH 8.0 + proteinase K	0.006	0.12	
		0.5 M EDTANa_2_ pH 8.0 + 20% SDS + 1 M Tris-HCl. 5 M NaCl + proteinase K	0.009	0.17	
No. 7	56 °C	20 μL proteinase K (0.25 mg/mL)	0.282	5.64	6.01 ± 0.52
		20 μL 0.5 M EDTANa_2_ pH 8.0 + 20 μL proteinase K (0.25 mg/mL)	0.319	6.38	
No. 8	56 °C	20 μL proteinase K (0.25 mg/mL)	0.72	14.31	14.92 ± 0.86
		20 μL 0.5 M EDTANa_2_ pH 8.0 + 20 μL proteinase K (0.25 mg/mL)	0.78	15.53	

**Table 2:** Results of aDNA extraction from 70 human teeth, using a non-destructive method that incorporates various purification techniques, are presented in chronological order.

No. of Sample (See List in Paragraph Section 2.1.)	Elution Temp.	Purification Method	Total Yield (μg)	aDNA Concentration (ng/μL)	Average aDNA Concentration ± SD	Results (bp)		
						100	200	300
No. 8	56 °C	QIAamp DNA Micro Kit (Qiagen)	0.716	14.31	14.92 ± 0.86	+	+	+
			0.777	15.53		+	+	+
No. 9	37 °C	DNA Extraction Kit FERMENTAS	0.002	0.03	0.04 ± 0.02	+	+	−
	56 °C	DNA Extraction Kit FERMENTAS	0.004	0.08		+	+	+
	37 °C	QIAamp DNA Micro Kit (Qiagen)	0.002	0.04		+	+	+
	56 °C	QIAamp DNA Micro Kit (Qiagen)	0.003	0.05		+	+	+
	37 °C	phenol–chloroform	0.001	0.02		+	+	−
	56 °C	phenol–chloroform	0.001	0.02		+	+	−
No. 10	37 °C	DNA Extraction Kit FERMENTAS	0.010	0.20	0.31 ± 0.13	+	+	−
	56 °C	DNA Extraction Kit FERMENTAS	0.027	0.53		+	+	+
	37 °C	QIAamp DNA Micro Kit (Qiagen)	0.020	0.40		+	+	−
	56 °C	QIAamp DNA Micro Kit (Qiagen)	0.020	0.40		+	+	+
	37 °C	phenol–chloroform	0.014	0.26		+	+	−
	56 °C	phenol–chloroform	0.018	0.35		+	+	−
No. 11	37 °C	DNA Extraction Kit FERMENTAS	0.003	0.05	0.027 ± 0.025	+	+	−
	56 °C	DNA Extraction Kit FERMENTAS	0.003	0.06		+	+	−
	37 °C	QIAamp DNA Micro Kit (Qiagen)	0.001	0.02		+	−	−
	56 °C	QIAamp DNA Micro Kit (Qiagen)	0.002	0.03		+	+	−
	37 °C	phenol–chloroform	0	0		+	−	−
	56 °C	phenol–chloroform	0	0		+	−	−
No. 12	37 °C	DNA Extraction Kit FERMENTAS	0.005	0.10	0.09 ± 0.02	+	+	+
	56 °C	DNA Extraction Kit FERMENTAS	0.006	0.12		+	+	+
	37 °C	QIAamp DNA Micro Kit (Qiagen)	0.005	0.09		+	+	+
	56 °C	QIAamp DNA Micro Kit (Qiagen)	0.005	0.10		+	+	+
	37 °C	phenol–chloroform	0.003	0.06		+	+	+
	56 °C	phenol–chloroform	0.003	0.06		+	+	+
No. 13	56 °C	QIAamp DNA Micro Kit (Qiagen)	0.076	1.52	1.11 ± 0.29	+	+	+
			0.052	1.03		+	+	+
			0.044	0.87		+	+	+
			0.050	1.0		+	+	+
No. 14	56 °C	QIAamp DNA Micro Kit (Qiagen)	0.017	0.33	0.21 ± 0.09	+	+	+
			0.011	0.22		+	+	+
			0.006	0.12		+	+	+
			0.009	0.17		+	+	+
No. 15	56 °C	QIAamp DNA Micro Kit (Qiagen)	0.092	1.83	-	+	+	+
No. 16	56 °C	QIAamp DNA Micro Kit (Qiagen)	0.282	5.64	6.01 ± 0.52	+	−	−
			0.319	6.38		+	−	−
No. 17	56 °C	QIAamp DNA Micro Kit (Qiagen)	0.002	0.04	0.035 ± 0.01	+	+	−
			0.002	0.03		+	+	−
No. 18	56 °C	QIAamp DNA Micro Kit (Qiagen)	0.001	0.01	-	+	+	−
No. 19	56 °C	QIAamp DNA Micro Kit (Qiagen)	0.469	9.37	-	+	+	−
			0.050	1.00	0.96 ± 1.00	+	+	−
			0.002	0.03		−	−	−
			0.004	0.08		−	−	−
			0.003	0.06		+	+	−
			0.003	0.05		+	−	−
			0.041	0.81		+	−	−
			0.025	0.49		+	−	−
			0.052	1.03		−	−	−
			0.163	3.25		−	−	−
			0.059	1.18		−	−	−
			0.055	1.09		−	−	−
			0.122	2.44		−	−	−
No. 20	56 °C	QIAamp DNA Micro Kit (Qiagen)	0.081	1.62	-	+	+	−
No. 21	56 °C	QIAamp DNA Micro Kit (Qiagen)	0.001	0.02	-	+	+	−
No. 22	56 °C	QIAamp DNA Micro Kit (Qiagen)	0.002	0.03	0.16 ± 0.13	+	−	−
			0.013	0.26		+	−	−
			0.018	0.35		+	−	−
			0.005	0.09		+	+	−
			0.005	0.09		+	+	−
No. 23	56 °C	QIAamp DNA Micro Kit (Qiagen)	0.003	0.05	0.04 ± 0.02	+	−	−
			0.003	0.05		−	−	−
			0.002	0.03		−	−	−
			0.001	0.01		−	−	−
No. 24	56 °C	QIAamp DNA Micro Kit (Qiagen)	0	0	-	−	−	−
No. 25	56 °C	QIAamp DNA Micro Kit (Qiagen)	0	0	0	+	−	−
			0	0		+	−	−
No. 26	56 °C	QIAamp DNA Micro Kit (Qiagen)	0	0	0	−	−	−

+,+,+ means complete results; +,+,− and +,−,− means partial results.

**Table 3:** The rate of success obtained in this paper using destructive (autopsy teeth) and non-destructive DNA isolation methods.

Number of Tested Samples (*N*)	Type of Sample	Results	Rate of Success
30	Autopsy teeth (fresh)	7 × FP, 19 × IR, 4 × NR	87%
6	Museal	4 × FP, 2 × IR,	100%
27	Museal tested after 20 years)	16 × FP, 11 × IR	100%
13	Archeology (1562–1620)	6 × IR, 7 × NR	54%
2	Archeology (60 years old)	2 × IR	100%
1	Archeology (World War II)	1 × IR	100%
1	Archeology (XV century)	1 × IR	100%
1	Archeology (XIII century)	1 × IR	100%
5	Archeology (XI century)	5 × IR	100%
4	Archeology (X century)	2 × IR, 2 × NR	50%
1	Archeology (V century)	1 × NR	0%
2	Archeology (4000 B.C.)	2 × IR	100%
1	Archeology (Neanderthal, molar tooth, dated about 50,000–100,000 years old)	1 × NR	0%

FP—full profile, IR—incomplete results, NR—no result.

## Data Availability

Data is unavailable due to ethical restrictions.
